# Establishment and Application of a Novel Protein Microarray for Serological Detection and Differentiation of Senecavirus A

**DOI:** 10.1155/tbed/5543555

**Published:** 2026-02-03

**Authors:** Dexin Li, Junhua Deng, Zenglin Wang, Yunjing Zhang, Yufang Li, Liying Hao, Kegong Tian, Xiangdong Li

**Affiliations:** ^1^ Jiangsu Co-innovation Center for Prevention and Control of Important Animal Infectious Diseases and Zoonoses, College of Veterinary Medicine, Yangzhou University, Yangzhou, 225009, China, yzu.edu.cn; ^2^ Luoyang Putai Biotechnology Co., Ltd., Luoyang, 471003, China; ^3^ National Research Center for Veterinary Medicine, Luoyang, 471003, China; ^4^ Joint International Research Laboratory of Agriculture and Agri-Product Safety, The Ministry of Education of China, Yangzhou University, Yangzhou, 225009, China, yzu.edu.cn

**Keywords:** DIVA, dual antibody detection, protein microarray, senecavirus A, tandem antigen

## Abstract

Senecavirus A (SVA) is an emerging swine pathogen that causes vesicular disease, which presents clinically indistinguishable signs from other vesicular diseases. To enable differentiation of infected from vaccinated animals (DIVA), we developed a novel protein microarray for dual serological detection of antibodies against SVA structural (VP2‐VP3‐VP1) and non‐structural (3AB‐3C) proteins. The assay was based on two novel His‐tagged tandem antigens, designed from immunodominant B‐cell epitopes, which were expressed in *Escherichia coli* (*E. coli*), purified, and spotted onto a poly(dimethylsiloxane) (PDMS) substrate. Results were quantified by spot gray values to calculate sample‐to‐positive (S/P) ratios, with cut‐off values set at S/P ≥0.651 for VP2‐VP3‐VP1 and S/P ≥0.607 for 3AB‐3C. The microarray successfully differentiated inactivated‐vaccine‐immunized animals (positive only for VP2‐VP3‐VP1) from live SVA‐challenged animals (positive for both antigens). In live SVA‐challenged pigs, seroconversion to the structural protein antigen VP2‐VP3‐VP1 occurred 4 days earlier than the non‐structural protein antigen 3AB‐3C, identifying it as a sensitive early diagnostic marker. Clinical validation demonstrated 97.5% concordance with the virus neutralization test (VNT), confirming the microarray as a reliable, high‐throughput tool for DIVA serological testing.

## 1. Introduction

Senecavirus A (SVA), the only member of the genus *Senecavirus* within the family *Picornaviridae*, was first identified in the United States in 2002 during cell culture propagation and initially designated as Seneca Valley virus‐001 (SVV‐001) [1]. It was later recognized as a novel oncolytic virus with potential antitumor applications [2]. SVA is now endemic in numerous countries worldwide, including Canada [3], the United States [1], Brazil [4], Colombia [5], Thailand [6], Vietnam [7], and China [8, 9]. SVA infection in pigs results in vesicular lesions that are clinically indistinguishable from those caused by other vesicular disease viruses, including foot‐and‐mouth disease virus (FMDV), vesicular stomatitis virus (VSV), vesicular exanthema of swine virus (VESV), and swine vesicular disease virus (SVDV) [10]. Due to this clinical similarity, laboratory‐based differential diagnosis is essential for distinguishing SVA from other vesicular diseases.

The SVA genome comprises a single‐stranded, positive‐sense RNA molecule ~7.2 kb in length and is non‐enveloped [2]. It contains a single open reading frame (ORF) flanked by 5′ and 3′ untranslated regions and a poly(A) tail. This ORF is translated into a polyprotein, which is subsequently cleaved into four structural and seven non‐structural proteins (NSPs) following the characteristic picornaviral L‐4‐3‐4 arrangement: L‐VP4‐VP2‐VP3‐VP1‐2A‐2B‐2C‐3A‐3B‐3C‐3D [2, 10]. Among the structural proteins, VP1, VP2, and VP3 are exposed on the capsid surface and serve as primary targets for neutralizing antibodies, whereas VP4 is located internally [2, 11]. Although VP1 is generally the most immunogenic protein in picornaviruses and is commonly used in vaccines [12, 13], recent studies have demonstrated that SVA infection elicits significantly strong antibody responses against VP2, indicating a unique immunogenic profile for this virus [14]. In contrast to structural proteins, NSPs are not incorporated into virions but are essential for viral replication and gene regulation [15]. The development of inactivated SVA vaccines has elevated the diagnostic importance of NSPs, which serve as ideal markers for distinguishing natural infection from vaccination [16]. Serological detection of antibodies against specific NSPs (particularly 3AB, 3C, and 3ABC) enables the DIVA, a strategy aligned with World Organization for Animal Health recommendations for FMDV [17–19].

Multiple serological methods are available for SVA detection, including indirect immunofluorescence assay (IFA) [14, 20], virus neutralization test (VNT) [20], immunohistochemistry assay (IHC) [21], and fluorescent microsphere immunoassays (FMIA) [11]. VNT remains the gold standard for detecting neutralizing antibodies due to its high specificity [22]. In contrast, enzyme‐linked immunosorbent assay (ELISA) is widely preferred for its simplicity, cost‐effectiveness, and high‐throughput [23]. Various ELISA formats have been developed, including indirect ELISAs based on recombinant VP1 [24], VP2 [14], VP3 [14], and 3AB [22, 25] proteins; competitive ELISAs using purified whole virus [20, 21, 23, 26]; double‐antigen sandwich ELISA [27]; and liquid‐phase blocking ELISA [28]. A key limitation of these conventional formats is that they primarily assess either vaccine‐induced immunity (via structural protein‐specific antibodies) or natural infection (via non‐structural protein responses), but not simultaneously. In contrast, emerging protein microarray technology enables the simultaneous multiplex detection of multiple antibodies in a single assay. It has been successfully applied in many cases—including multiplex detection of avian influenza virus antibodies [29], serodiagnosis of pseudorabies virus proteins [30], and high‐throughput viral epitope screening [31, 32].

In this study, we developed a high‐throughput protein microarray on a PDMS substrate for the serological differentiation of SVA‐infected and vaccinated animals. To achieve this, we rationally designed two novel tandem recombinant antigens (His‐VP2‐VP3‐VP1 and His‐3AB‐3C) based on immunodominant B‐cell epitopes within SVA structural (VP1, VP2, VP3) and non‐structural (3AB, 3C) proteins. These antigens were efficiently expressed in *Escherichia coli* (*E. coli*) and used for the microarray. The resultant microarray enables simultaneous quantification of antibodies against both the structural (VP2‐VP3‐VP1) and non‐structural (3AB‐3C) proteins in a single assay. Critically, evaluation using sera from inactivated‐vaccine‐immunized and live SVA‐challenged pigs confirmed that the assay reliably distinguishes vaccinated animals (positive only for VP2‐VP3‐VP1) from infected animals (positive for both antigens). This microarray provides an efficient tool for supporting SVA differential diagnosis and large‐scale surveillance programs.

## 2. Materials and Methods

### 2.1. Viruses, Cells, Antibodies, and Serum Samples

The SVA strain CH‐HNXC was provided by the National Research Center for Veterinary Medicine. Instituto Biologico‐Rim Suino‐2 (IBRS‐2) cells, used for virus propagation and neutralization assays, were cultured in Dulbecco’s modified Eagle’s medium (DMEM; Gibco, Langley, OK, USA) supplemented with 8% heat‐inactivated fetal bovine serum (FBS; Biological Industries, Beit HaEmek, Israel) and maintained at 37°C under 5% CO_2_.

The following antibodies were used: horseradish peroxidase (HRP)‐conjugated goat anti‐pig IgG (H + L) secondary antibody (Abcam, Waltham, MA, USA) and pig IgG (Luoyang Putai Biotechnology Co., Ltd., Luoyang, China).

A defined panel of swine serum samples was utilized for assay validation and evaluation. The following subsets were derived from our previously described serum collection [33] and were applied to new analyses in this study: (i) 200 samples (100 VNT‐positive and 100 VNT‐negative) for determining cut‐off values; and (ii) two longitudinal series from inactivated‐vaccine‐immunized (weeks 0−4 post‐vaccination) and live SVA‐challenged (days 0−12 post‐challenge) pigs to evaluate diagnostic sensitivity and DIVA capacity, respectively. Additionally, 40 new clinical samples, not included in the prior study, were incorporated for the independent assessment of diagnostic sensitivity. All serum samples were characterized by prior testing for neutralizing antibodies.

To assess analytical specificity, antibody‐positive serum samples against foot‐and‐mouth disease virus (FMDV) serotypes A and O were included. These sera were provided by the National Research Center for Veterinary Medicine. Porcine reproductive and respiratory syndrome virus (PRRSV), pseudorabies virus (PRV), porcine circovirus 2 (PCV2), porcine circovirus 3 (PCV3), and classical swine fever virus (CSFV) standard antibody‐positive sera were provided by Beijing Sino‐science Gene Technology Co., Ltd. (Beijing, China). African swine fever virus (ASFV) standard antibody‐positive serum was purchased from the China Institute of Veterinary Drug Control (Beijing, China). Furthermore, FMDV serotype O‐ and A‐specific antibody ELISA kits (Luoyang Putai Biotechnology Co., Ltd., Luoyang, China) were used to confirm the status of FMDV‐positive sera.

### 2.2. Screening of Dominant Epitopes and Plasmid Construction

To identify potential linear B‐cell epitopes, the antigenic indices of SVA proteins (VP1, VP2, VP3, 3AB, and 3C) were predicted using DNAStar Protean software (version 7.1) [34] and the Bepipred Linear Epitope Prediction 2.0 tool from the Immune Epitope Database (IEDB) Analysis Resource [35]. These predictions were then integrated with a comprehensive set of published epitope data to select the final antigenic regions. The incorporated epitopes included for VP1, ^21^GELAAP^26^ [13]; for VP2, ^12^DRVITQT^18^, ^71^WTKAVK^76^, ^98^GGAFTA^103^, ^150^KSLQELN^156^, ^248^YKEGAT^253^ [13], ^156^NEEQWV^161^, ^262^VRPTSPYFN^270^ [36], ^153^QELNEE^158^ [37], and ^271^GLRNRFTTGTDEEQ^284^ [38]; for VP3, ^192^GWFSLHKLTK^201^ [39]; for 3AB ^5^NDDTPVDEALGR^16^ [35], ^90^NAYDGPKKNS^100^ [40], and ^75^QEETEG^80^ [41]; and for 3C ^75^FTHHGLPTDL^85^ and ^95^DQMPARNSRV^105^ [40]. Based on this integrated analysis, the following regions were selected (Figure 1A): three in VP1 (aa 5−100, 145−177, and 199−261), three in VP2 (aa 2−103, 141−198, and 211−284), four in VP3 (aa 7−32, 57−81, 135−153, and 173−236), two in 3AB (aa 1−36 and 69−105), and three in 3C (aa 4−29, 48−112, and 138−174).

Figure 1Design, production, and immunological validation of recombinant SVA antigens. (A) Schematic of the tandem antigen constructs and their corresponding expression plasmids. (B, C) The expressed proteins were analyzed by (B) SDS‐PAGE, confirming size and purity, and (C) Western blot, demonstrating specific immunoreactivity with SVA‐positive swine serum. Lanes: M, protein molecular weight marker; 1, VP1 (~82 kDa); 2, VP2 (~36 kDa); 3, VP3 (~28 kDa); 4, 3AB (~70 kDa); 5, 3C (~24 kDa); 6, VP2‐VP3‐VP1 (~73 kDa); 7, 3AB‐3C (~28 kDa). The lane order is identical in panels B and C.

**Figure:**
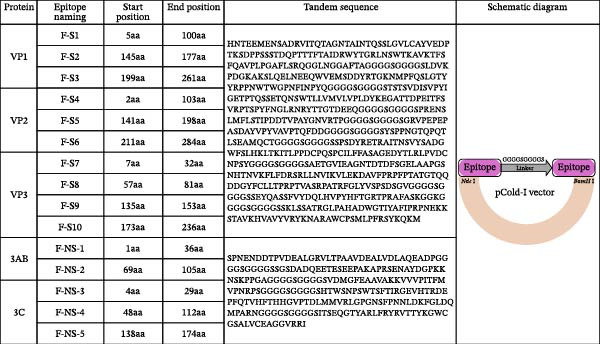
(A)

**Figure:**
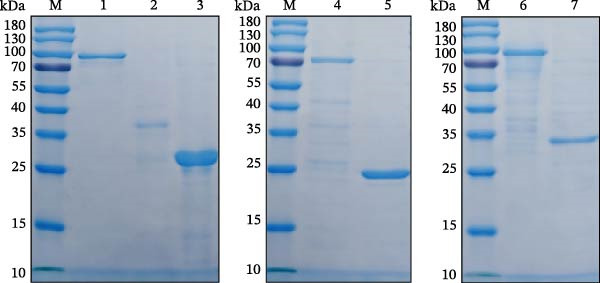
(B)

**Figure:**
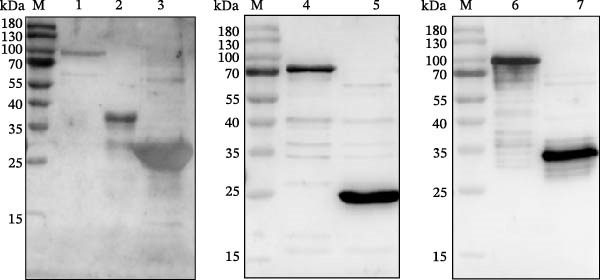
(C)

To generate the tandem antigens, selected epitopes from VP1, VP2, and VP3 were connected via synthetic glycine‐serine linkers (GGGGS)_2_ to form a recombinant gene, designated *VP2-VP3-VP1*. Similarly, epitopes from 3AB and 3C were linked to construct the *3AB-3C* gene. Both synthetic genes were codon‐optimized for *E. coli* expression, commercially synthesized, and cloned into the pCold‐I vector using *Nde*I/*BamH*I restriction sites (GenScript, Nanjing, China).

For individual protein expression, the genes encoding *VP1*, *VP2*, *VP3*, *3AB*, and *3C* were cloned into the following vectors: pCold‐TF (for VP1 and 3AB), pET‐28a (for VP2), or pCold I (for VP3 and 3C). Protein expression was subsequently carried out in *E. coli* Trans BL21(DE3) plysS (TransGen Biotech, Beijing, China). All primer sequences used for cloning are listed in Table S1.

### 2.3. Expression in *E. coli* and Purification

The recombinant plasmids were transformed into *E. coli* BL21(DE3) competent cells. Transformed cells were cultured in Luria‐Bertani (LB) medium at 37°C with shaking until the OD_600_ reached 0.4−0.6. For proteins expressed from the pCold‐I and pCold‐TF vectors (VP1, VP3, 3AB, 3C, VP2‐VP3‐VP1, and 3AB‐3C), the culture medium was supplemented with 0.1 mg/mL ampicillin, and protein expression was induced with 0.2 mM isopropyl‐β‐D‐1‐thiogalactopyranoside (IPTG; Solarbio, Beijing, China) at 16°C for 16−20 h. For the pET‐28a‐VP2 construct, the medium was supplemented with 0.05 mg/mL kanamycin, and expression was induced with 1 mM IPTG at 37°C for 5−6 h.

After induction, cells were harvested by centrifugation and lysed by ultrasonication on ice. The lysates were centrifuged at 12,000 × *g* for 20 min at 4°C to separate soluble and insoluble fractions. SDS‐PAGE was used to analyze the supernatant and pellet to determine the solubility of the expressed proteins.

His‐tagged recombinant proteins were purified under native (for soluble proteins) or denaturing (for insoluble proteins) conditions using High‐Affinity Ni‐NTA Resin (GenScript USA Inc., Piscataway, NJ, USA). For insoluble proteins, the resin‐bound complexes were washed three times with a denaturing binding buffer (100 mM NaH_2_PO_4_, 10 mM Tris‐Cl, 10 mM imidazole, 8 M urea, pH 8.0), and target proteins were eluted with denaturing elution buffer containing 250 mM imidazole and 8 M urea. Purification of soluble proteins was performed under native conditions. After the target protein was bound to the Ni‐NTA resin, a series of washes with native buffer (50 mM NaH_2_PO_4_, 50 mM NaCl, 10 mM imidazole, pH 8.0) were applied to remove non‐specifically bound contaminants. The purified protein was then competitively eluted by increasing the imidazole concentration to 250 mM in the elution buffer.

### 2.4. SDS‐PAGE and Western Blot

Protein samples were separated by SDS‐PAGE and transferred onto a polyvinylidene fluoride (PVDF) membrane (Millipore, Darmstadt, Germany). The membrane was blocked with 5% bovine serum albumin (BSA; Solarbio, Beijing, China) for 2 h at room temperature and subsequently incubated with SVA‐positive swine serum (1:1000 dilution in blocking buffer) for 2 h at room temperature. After five washes with PBST (phosphate‐buffered saline (PBS) containing 0.05% Tween‐20), the membrane was probed with an HRP‐conjugated goat anti‐pig IgG (H + L) secondary antibody (1:10,000 dilution) for 1 h at room temperature. Following extensive washing, immunoreactive bands were visualized using Clarity Western ECL Substrate (NCM Biotech Co. Ltd., Suzhou, China) and imaged with a VILBER Fusion FX7 system (Vilber Lourmat, Collégien, France).

### 2.5. VNT

The VNT was conducted using a fixed virus‐diluted serum approach. Briefly, test sera were first inactivated by incubation at 56°C for 30 min. Subsequently, two‐fold serial dilutions of the inactivated sera were prepared in a 96‐well plate. To these serum dilutions, we added an equal volume of a challenge virus standard containing 200 50% tissue culture infective doses (TCID_50_) of SVA per 50 μL. The plate was then incubated for 1 h at 37°C to facilitate the virus‐neutralizing antibody reaction. Subsequently, 50 μL of an IBRS‐2 cell suspension (2.5 × 10^4^ cells/well in DMEM supplemented with 8% fetal bovine serum) was added to each well. The plate was incubated at 37°C under 5% CO_2_ and examined daily for cytopathic effect (CPE) for 3 days. The assay included the following controls: normal cells (cell control), serum‐only (serum control), and virus‐only (virus control). The test was considered valid only if the virus control exhibited complete CPE while the cell and serum controls remained unaffected. The neutralizing antibody titer was the reciprocal of the highest serum dilution inhibiting CPE in ≥50% of the cells.

### 2.6. Protein Microarray Preparation

The nanomembrane, fabricated with surface‐grafted poly(dimethylsiloxane) (PDMS) brushes via surface‐initiated polymerization using initiator‐integrated PDMS (iPDMS), was activated by complete immersion in a solution containing 1‐(3‐dimethylaminopropyl)‐3‐ethylcarbodiimide (EDC) and N‐hydroxysulfosuccinimide (NHS) for 30 min. The membrane was then rinsed three times with ultrapure water and air‐dried. All fabrication steps were performed by Luoyang Putai Biotechnology Co., Ltd. (Luoyang, China).

### 2.7. Establishment of the Protein Microarray Detection Method

The protein microarray was established by spotting purified antigens onto an activated PDMS substrate at five concentrations (0.05 to 0.8 mg/mL). Each subarray included pig IgG (0.005, 0.01, and 0.02 mg/mL) and spotting buffer (2.5 nL) as internal quality controls. After spotting, the microarrays were blocked with 1% BSA for 2 h at room temperature, air‐dried at 20°C−25°C for 16−20 h, and stored at 2°C−8°C until use.

Assay conditions were optimized using a checkerboard titration approach. Initial optimization was performed under a baseline protocol (30 min incubation for serum and secondary antibody at 37°C with 500 rpm shaking; 15 min 3,3′,5,5′‐tetramethylbenzidine (TMB) development under the same conditions) to determine the optimal antigen coating concentration, serum dilution (1:25, 1:50, 1:100), and secondary antibody dilution (1:5000 to 1:40,000). Subsequent optimization evaluated blocking solutions (1% BSA, 5% skim milk, 1% ovalbumin), blocking conditions (2 h at 25°C, 2 h at 37°C, or 12 h at 4°C), and incubation times for serum and secondary antibody (30, 45, or 60 min at 37°C).

The optimal condition for each parameter was selected based on the highest positive‐to‐negative (P/N) value, ensuring a strong signal from positive samples and a low background from negative controls. The finalized protocol was validated using five SVA antibody‐positive and three antibody‐negative serum samples, with all experiments performed in triplicate. Luoyang Putai Biotechnology Co., Ltd. supplied all reagents, including spotting buffers, blocking solutions, washing liquid, serum diluent, antibody diluent, and TMB.

### 2.8. Determination of the Cut‐Off Values

Cut‐off values for the SVA‐VP2‐VP3‐VP1 and SVA‐3AB‐3C antigens were determined separately using a panel of 200 well‐characterized serum samples, comprising 100 VNT‐confirmed SVA‐positive and 100 SVA‐negative samples. Sample‐to‐positive (S/P) ratios were calculated from the grayscale values using the following formula: (sample grayscale value − negative control grayscale value)/(positive control grayscale value − negative control grayscale value). For this calculation, the negative control was serum from specific pathogen‐free (SPF) swine, confirmed to be SVA antibody‐negative, and the positive control was serum from an experimentally SVA‐infected swine, which had been confirmed as positive by virus neutralization assay.

For each antigen, receiver operating characteristic (ROC) curve analysis was performed on the S/P ratios using GraphPad Prism 8.0 to evaluate its diagnostic accuracy in distinguishing positive from negative sera and to determine the optimal antigen‐specific cut‐off value. The optimal cut‐off value and corresponding sensitivity and specificity were selected by maximizing Youden’s index [42, 43].

### 2.9. Determination of the Sensitivity, Specificity, Repeatability and Reproducibility

Analytical sensitivity was determined by testing a two‐fold serial dilution (1:2 to 1:128) of a known SVA antibody‐positive control serum under optimized microarray conditions. The endpoint titer was defined as the one with the highest dilution yielding a positive result.

Diagnostic sensitivity was evaluated using 40 clinical serum samples with predetermined neutralizing antibody status by VNT. A sample was considered positive if it reacted positively to at least one antigen on the microarray. The results were then compared against the VNT benchmark to calculate agreement.

Analytical specificity (cross‐reactivity) was assessed by testing the microarray against antibody‐positive sera for major swine pathogens (FMDV, PRRSV, PRV, PCV2, PCV3, CSFV, ASFV). Three positive samples per pathogen were tested, except for FMDV serotypes O and A (*n* = 20). Further details on these sera are provided in Section 2.1.

Assay precision, including intra‐assay repeatability and inter‐assay reproducibility, was evaluated using three antisera with strong, moderate, and weak positive reactivity. Intra‐assay variability was determined by testing each sample eight times within a single run. Inter‐assay reproducibility was assessed by testing each sample eight times across three independent plates. Precision was quantified by calculating the coefficient of variation (CV = standard deviation (SD)/average value (x̄)) for each antiserum.

### 2.10. Statistical Analysis

All statistical analyses and graphical generation were performed using GraphPad Prism version 8.0 (GraphPad Software, San Diego, CA, USA). All graphs were generated using GraphPad Prism. Data were analyzed by Student’s *t*‐test.  ^∗^ *p* < 0.05 was considered significant, with specific levels indicated as  ^∗∗∗^ *p* < 0.001,  ^∗∗^ *p* < 0.01, and  ^∗^ *p* < 0.05.

## 3. Results

### 3.1. Analysis of Dominant Epitopes and Expression and Purification of Recombinant Proteins

The immunodominant epitopes were identified, and tandem antigens were constructed as described in Section 2.2, as shown schematically in Figure 1A. Using an *E. coli* prokaryotic expression system, we successfully obtained high‐yield soluble expression of recombinant VP1, 3AB, 3C, and the tandem epitope protein 3AB‐3C. In contrast, VP2, VP3, and the tandem epitope protein VP2‐VP3‐VP1 were expressed as insoluble forms as inclusion bodies. All recombinant proteins were purified by Ni‐NTA affinity chromatography under native (soluble proteins) or denaturing (inclusion bodies) conditions. The purity and molecular weights of the purified proteins were confirmed by SDS‐PAGE, each exhibiting a single band at the expected size (Figure 1B). Additionally, Western blot analysis using sera from SVA‐positive pigs demonstrated specific immunoreactivity for each recombinant protein (Figure 1C).

### 3.2. Establishment, Optimization, and Cut‐Off Value Determination of the Protein Microarray

We optimized assay conditions using checkerboard titration, selecting parameters that yielded high P/N values and distinct separation in grayscale values between positive and negative control sera. Optimal conditions were determined: serum dilution at 1:50 and antigen spotting concentration at 0.5 mg/mL for both the SVA‐VP2‐VP3‐VP1 and 3AB‐3C tandem proteins. The HRP‐conjugated goat anti‐pig IgG secondary antibody performed optimally at 1:10,000 and 1:20,000 dilutions for the respective antigens (Figure 2A, B). Since no significant differences were observed among various blocking buffers or conditions (Figure 2C, D), we selected 1% BSA with incubation at 37°C for 2 h. Likewise, serum and secondary antibody incubation times of 30 min and 45 min showed no notable difference (Figure 2E); the shorter 30 min protocol was therefore adopted for both steps to enhance throughput. To ensure consistency across the co‐spotted microarray, a unified secondary antibody dilution of 1:10,000 was used for both recombinant antigens.

Figure 2Optimization of assay conditions and determination of diagnostic cut‐off values for the SVA protein microarray. (A, B) Checkerboard titration to determine the optimal antigen spotting concentration and secondary antibody dilution for the recombinant VP2‐VP3‐VP1 and 3AB‐3C proteins. Data are presented as heatmaps of P/N values. The line graph represents the corresponding P/N values. (C) Evaluation of different blocking solutions. (D) Optimization of blocking conditions. (E) Screening of optimal incubation times for serum and HRP‐conjugated secondary antibody. (F, G) Assessment of the diagnostic performance of the protein microarray for the VP2‐VP3‐VP1 (F) and 3AB‐3C (G) antigens. Left panels: ROC curves with AUC values indicating overall accuracy. Right panels: Dot plots of S/P ratios for known positive and negative serum samples. The dashed horizontal line denotes the optimal cut‐off value determined by maximizing Youden’s index. Student’s *t*‐test evaluated differences,  ^∗∗∗^ *p* < 0.001,  ^∗∗^ *p*  < 0.01,  ^∗^ *p* < 0.05, ns: not significant.

**Figure:**
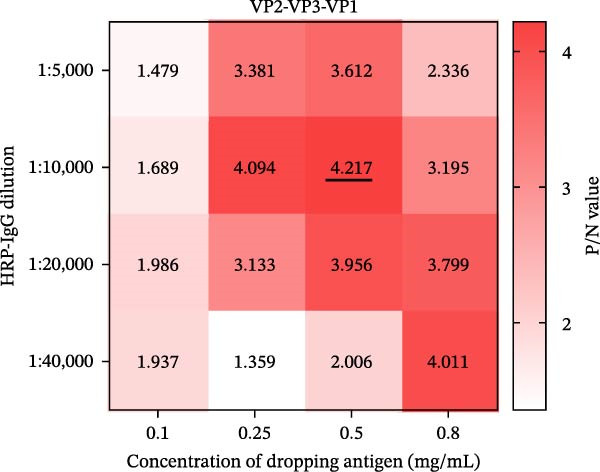
(A)

**Figure:**
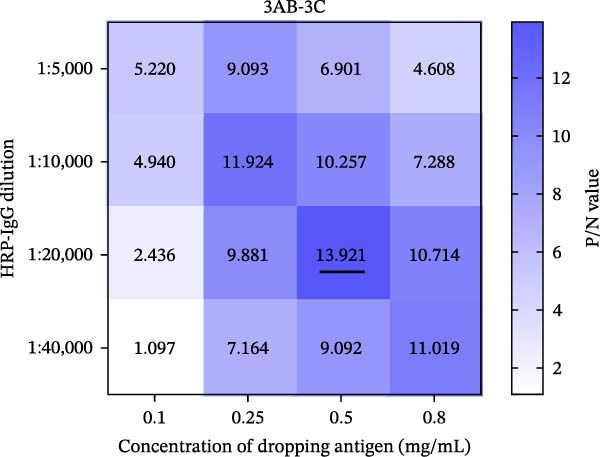
(B)

**Figure:**
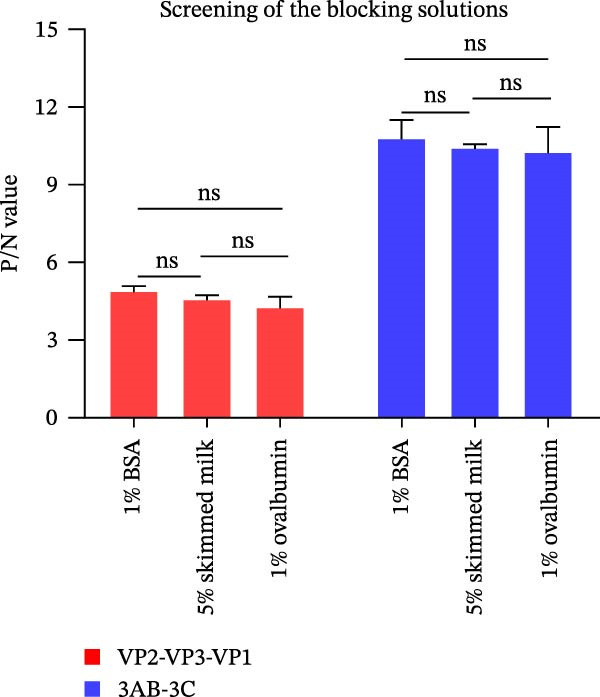
(C)

**Figure:**
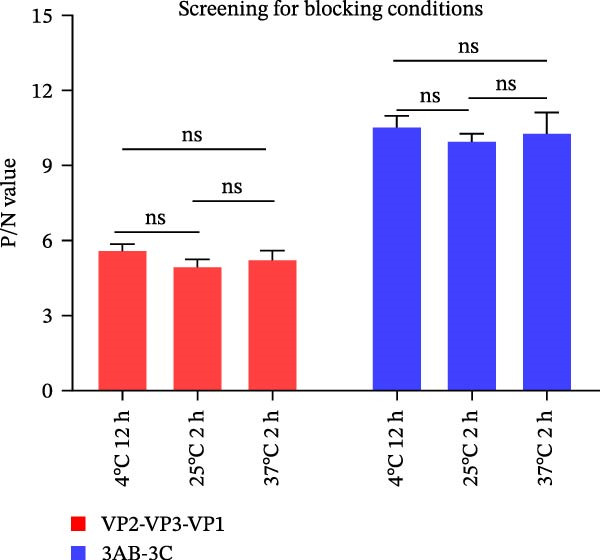
(D)

**Figure:**
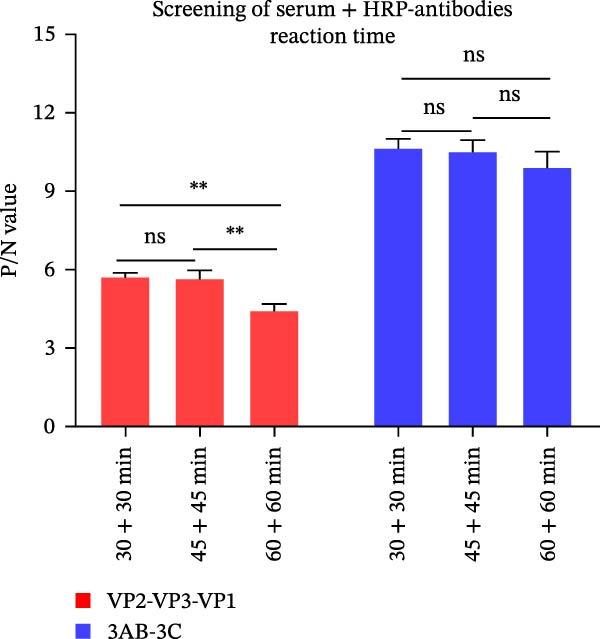
(E)

**Figure:**
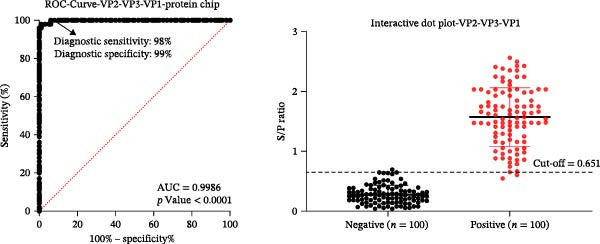
(F)

**Figure:**
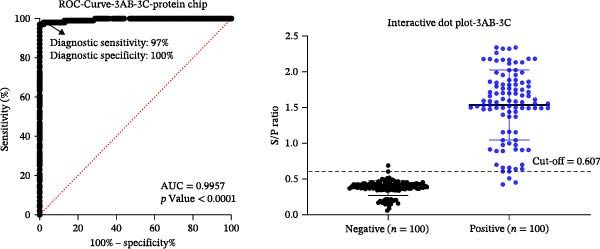
(G)

Next, we evaluated the performance of the optimized protein microarray using a panel of 200 swine serum samples, which included 100 VNT‐confirmed SVA‐positive and 100 SVA‐negative sera. All samples were tested, and S/P ratios were calculated. ROC curve analysis determined the optimal cut‐off values. The area under the curve (AUC) was 0.9986 (*p* < 0.0001; 95% confidence interval (CI): 0.9965−1.000) for the VP2‐VP3‐VP1 antigen and 0.9957 (*p* < 0.0001; 95% CI: 0.9893−1.000) for the 3AB‐3C antigen (Figure 2F, G, left panels). Using the maximum Youden’s index in GraphPad Prism 8.0, we established cut‐off values of 0.651 for VP2‐VP3‐VP1 and 0.607 for 3AB‐3C, achieving high diagnostic sensitivity and specificity (Figure 2F, G, right panels).

Additionally, we developed a separate protein microarray based on individual SVA structural (VP1, VP2, VP3) and non‐structural (3AB, 3C) proteins, which was also optimized by checkerboard titration (Figure S1). The optimal spotting concentrations were 0.5 mg/mL for VP1, VP2, 3AB, and 3C, and 0.25 mg/mL for VP3. This array used a serum dilution of 1:25, a secondary antibody dilution of 1:10,000, and serum and secondary antibody incubations were conducted at 37°C for 30 min each. Blocking was performed with 1% BSA at 37°C for 2 h, and TMB substrate incubation lasted 15 min at 37°C in the dark.

### 3.3. Analytical Validation: Immunoreactivity, Sensitivity, and Specificity

We assessed the immunoreactivity of five recombinant SVA proteins (the structural proteins VP1, VP2, and VP3 and the NSPs 3AB and 3C) and two tandem antigens (VP2‐VP3‐VP1 and 3AB‐3C) using protein microarrays. Based on P/N values, the VP2‐VP3‐VP1 tandem antigen showed higher immunoreactivity than the individual structural proteins, and the 3AB‐3C tandem antigen showed higher immunoreactivity than the single NSPs (Figure 3A).

Figure 3Analytical performance of the SVA protein microarray. (A) Immunoreactivity of individual and tandem SVA antigens. Red bars indicate the mean gray values of SVA‐positive sera (*n* = 5) from three independent assays. Gray bars indicate the mean gray values of SVA‐negative sera (*n* = 3) from three independent assays. The line graph depicts the corresponding P/N values. (B) Analytical sensitivity was determined by two‐fold serial dilution (1:2 to 1:128) of a high‐titer SVA‐positive serum. (C) Assessment of cross‐reactivity with FMDV serotype O/A‐positive sera (*n* = 20). The left Y‐axis shows FMDV antibody percentage inhibition (values from commercial ELISA kits), and the right Y‐axis shows S/P ratios for the SVA antigens. (D) Cross‐reactivity of SVA tandem antigens with antisera against other swine pathogens (PRRSV, PRV, PCV2, PCV3, ASFV, CSFV). The horizontal dashed line in panels B−D indicates the assay‐specific cut‐off value.

**Figure:**
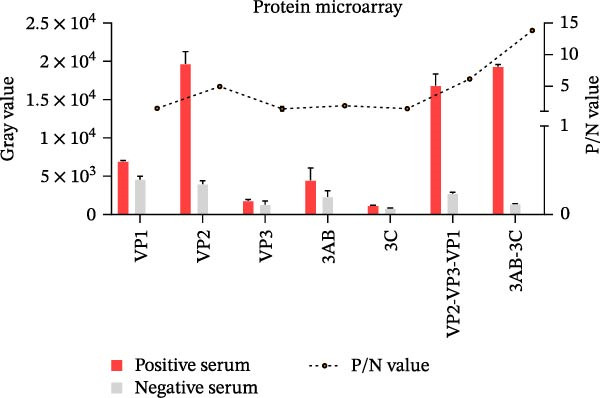
(A)

**Figure:**
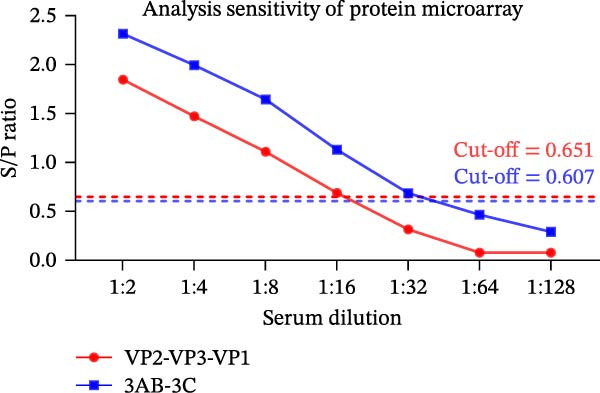
(B)

**Figure:**
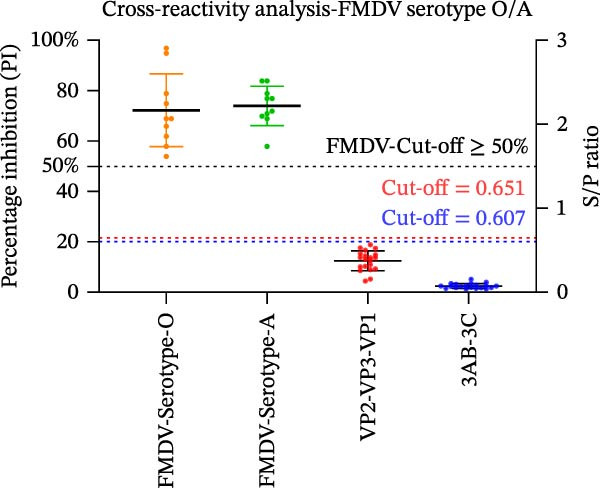
(C)

**Figure:**
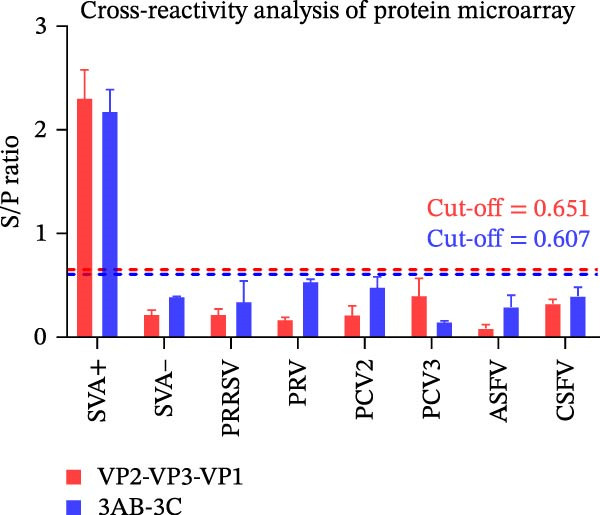
(D)

We determined analytical sensitivity by serially diluting a high‐titer positive serum. The assay based on VP2‐VP3‐VP1 detected antibodies at a dilution of 1:16, while the 3AB‐3C‐based assay detected antibodies at a dilution of 1:32 (Figure 3B).

To evaluate specificity, we tested sera positive for FMDV serotype O/A (*n* = 20; ELISA‐confirmed). Neither tandem antigen showed any cross‐reactivity (Figure 3C). Furthermore, both antigens showed no reactivity against sera positive for PRRSV, PRV, PCV2, PCV3, ASFV, or CSFV (Figure 3D), confirming high specificity for SVA antibody detection.

### 3.4. Evaluation of the Repeatability and Reproducibility

We evaluated the repeatability (intra‐assay variation) and reproducibility (inter‐assay variation) of the protein microarray assay for the VP2‐VP3‐VP1 and 3AB‐3C antigens by calculating the CV. Both antigens exhibited low CV values, indicating superior assay performance (Table 1). For the VP2‐VP3‐VP1 antigen, the intra‐ and inter‐assay CVs ranged from 3.8% to 9.5% and from 6.6% to 8.6%, respectively. Similarly, the 3AB‐3C antigen showed intra‐ and inter‐assay CVs of 3.4% to 8.7% and 4.6% to 9.1%, respectively. The consistently low CV values demonstrate high assay repeatability and reproducibility, supporting the reliability of this protein microarray for SVA serodiagnosis.

**Table 1:** Repeatability and reproducibility of the established protein microarray.

SVA antisera	Intra‐assay						Inter‐assay					
	VP2‐VP3‐VP1			3AB‐3C			VP2‐VP3‐VP1			3AB‐3C		
	x̄	SD	CV	x̄	SD	CV	x̄	SD	CV	x̄	SD	CV
Strongly positive	2.247	0.085	3.8%	2.267	0.076	3.4%	2.357	0.155	6.6%	2.097	0.186	8.9%
Moderately positive	1.816	0.116	6.4%	1.487	0.060	4.1%	1.863	0.151	8.1%	1.502	0.069	4.6%
Weakly positive	0.863	0.082	9.5%	0.702	0.061	8.7%	0.791	0.068	8.6%	0.723	0.066	9.1%

### 3.5. Diagnostic Sensitivity and DIVA Potential of the Established Microarray

The diagnostic performance and DIVA potential of the protein microarray were evaluated using sera from two independent animal studies. Serum samples from pigs in the inactivated vaccine immunization experiment and the live virus challenge experiment were analyzed separately by VNT and protein microarray, with the results shown in Figure 4A−F, respectively.

Figure 4Serological profiling and DIVA potential of the SVA protein microarray in the inactivated vaccine and live virus challenge models. (A) Virus neutralization test (VNT) results for inactivated‐vaccine‐immunized pigs. (B, C) Microarray‐measured IgG antibody kinetics against the (B) structural (VP2‐VP3‐VP1) and (C) non‐structural (3AB‐3C) tandem antigens in inactivated‐vaccine‐immunized pigs. (D) VNT results for live SVA‐challenged pigs. (E, F) IgG antibody kinetics against the (E) VP2‐VP3‐VP1 and (F) 3AB‐3C antigens in live SVA‐challenged pigs. (G) Array layout (left) and a representative chemiluminescent image (right). The three QC spots contain pig IgG and serve as positive controls and alignment markers; the Buffer spot contains diluent as a negative control. A valid assay requires all QC spots to be positive and the Buffer spot to be negative. Key samples: 1, negative control serum; 2, serum from an inactivated‐vaccine‐immunized pig (reactive only to VP2‐VP3‐VP1); 3, serum from a live SVA‐challenged pig (reactive to both antigens). The dashed horizontal line in panels denotes the cut‐off value.

**Figure:**
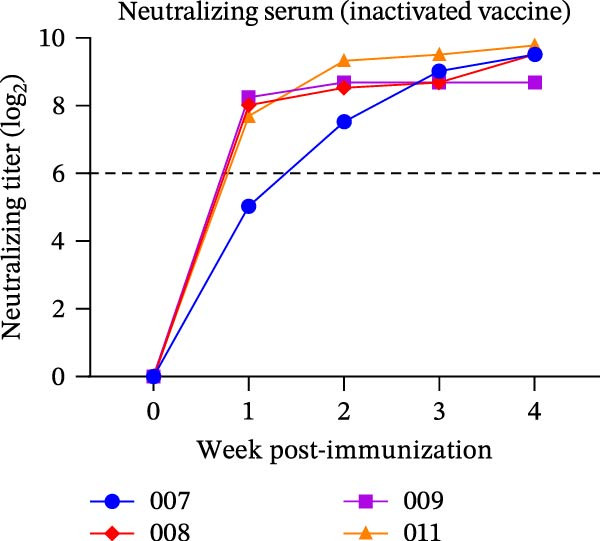
(A)

**Figure:**
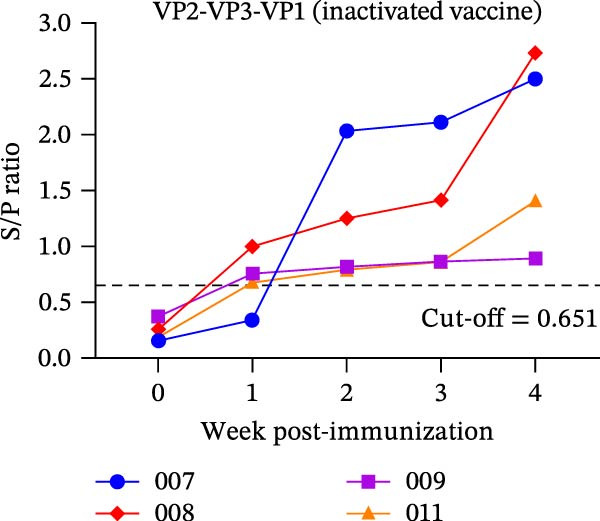
(B)

**Figure:**
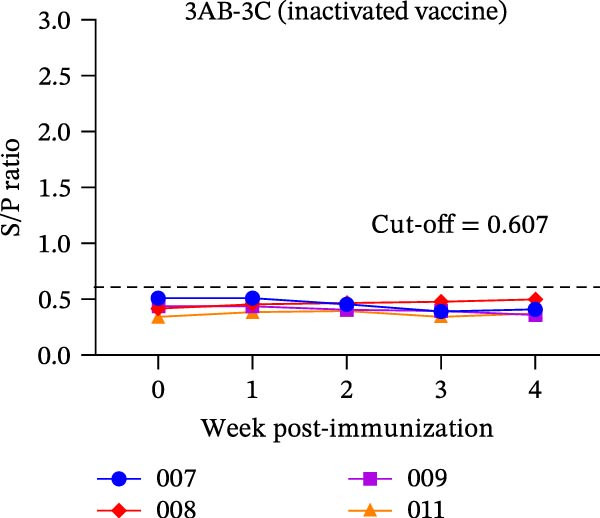
(C)

**Figure:**
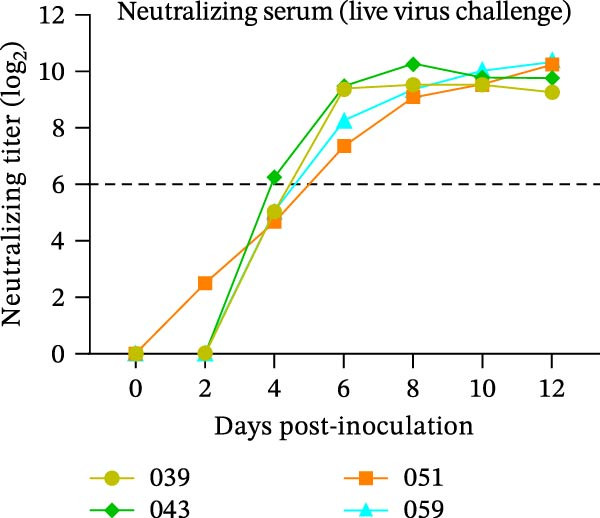
(D)

**Figure:**
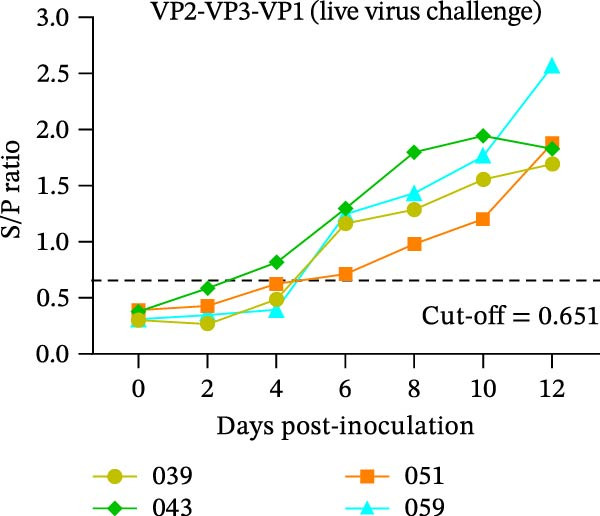
(E)

**Figure:**
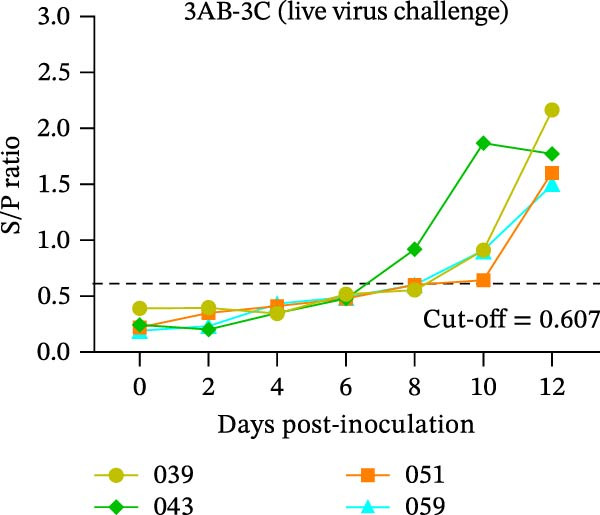
(F)

**Figure:**
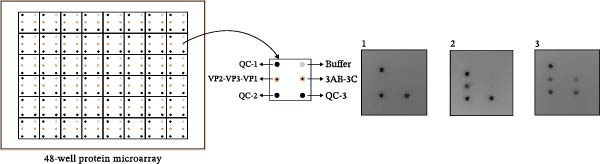
(G)

In the inactivated‐vaccine‐immunized pigs, IgG antibodies against the structural tandem antigen (VP2‐VP3‐VP1) seroconverted in synchrony with neutralizing antibodies. Pig #007 seroconverted at week 2, while pigs #008, #009, and #011 all seroconverted within the first week post‐immunization (Figure 4A, B). Notably, IgG antibodies against the non‐structural tandem antigen (3AB‐3C) remained undetectable in all vaccinated animals throughout the study (Figure 4C), confirming its ideal DIVA antigen characteristics.

In the live SVA‐challenged pigs, both antigens elicited antibody responses. The response to the structural antigen (VP2‐VP3‐VP1) was rapid and synchronous with neutralizing antibodies, with pig #043 seroconverting by day 4 and the others by day 6 (Figure 4D, E). In contrast, seroconversion to the non‐structural antigen (3AB‐3C) was significantly delayed, occurring on day 8 (pig #043) or day 10 (remaining pigs) post‐challenge (Figure 4F), highlighting the distinct temporal dynamics of the antibody response during infection.

A representative microarray image is presented in Figure 4G, depicting results for a negative control (1), serum from an inactivated‐vaccine‐immunized pig (2), and a positive serum from a live virus challenge (3).

To further validate the assay’s performance, we assessed its diagnostic sensitivity against a panel of 40 clinical sera (Table 2). Compared to the VNT benchmark, the microarray demonstrated 94.7% positive agreement (18/19), 100% negative agreement (21/21), and an overall concordance of 97.5%. The microarray did not detect one VNT‐positive sample.

**Table 2:** Diagnostic performance of protein microarray against the VNT benchmark for detecting SVA antibodies in clinical samples (*n* = 40).

Detection methods		Virus neutralization test		
		Positive	Negative	Total
Protein microarray	Positive	18	0	18
	Negative	1	21	22
	Total	19	21	40
Agreement rate (%)		94.7%	100%	97.5%

## 4. Discussion

SVA poses a significant threat to the global pig industry, causing clinical signs indistinguishable from those of foot‐and‐mouth disease (FMD) and vesicular stomatitis (VS), including vesicles on the snout and coronary bands, lameness, and fever [11]. This similarity complicates differential diagnosis and impedes effective FMD control strategies [10]. Although several serological assays are available for SVA detection, a practical tool for simultaneously assessing vaccine‐induced immunity and detecting natural infection is still lacking.

While ELISA remains the most widely used serodiagnosis method due to its high‐throughput capacity, it is generally limited to single‐antigen detection. In contrast, protein microarray technology allows for simultaneous multi‐antigen and multi‐sample analysis, enabling comprehensive serological profiling [29]. The protein microarray, also referred to as a “0 + X” nanomembrane chip, which offers key advantages: its near‐zero nonspecific background (“0”) enhances sensitivity and specificity, while flexible covalent conjugation of multiple antigens (“X”) ensures stable, high‐throughput multiplexing [30]. It should be noted that the use of a uniform sample and detection conditions (including a standardized serum dilution and a consistent secondary antibody concentration), which is necessary for assay consistency, may not represent the optimal detection condition for every individual antigen‐antibody pair. In this study, we developed a novel protein microarray assay on an PDMS membrane chip for the parallel detection of antibodies against SVA structural and NSPs. This platform facilitates high‐throughput evaluation of immune responses and supports a DIVA strategy. Our microarray exhibited 97.5% concordance with the VNT, confirming its high diagnostic reliability and efficiency for SVA serodiagnosis.

This DIVA concept is well‐established, notably with NSPs of FMDV [17, 44], and has been successfully applied to SVA using 3AB [22, 25]. Current SVA ELISA antigens primarily include individual recombinant proteins [14, 27], virus‐like particles (VLPs) [26], and inactivated virus [20, 21, 23]. However, assays relying on a single recombinant protein often provide incomplete epitope coverage, increasing the risk of false‐negative results [45]. Although VLPs and inactivated virus present a broader array of conformational and linear epitopes [28], their practical application can be hampered by batch‐to‐batch variations in purity, which may compromise assay reproducibility and reliability. Previous studies have shown that there is no significant difference in ELISA performance of prokaryotic and eukaryotic expression of SVA VP1 and 3AB [22, 24], and some strategies have utilized immunodominant epitopes for serodiagnosis [38, 45].

Therefore, we designed two tandem antigens, VP2‐VP3‐VP1 and 3AB‐3C, through integrated bioinformatic epitope prediction and literature review. These multiepitope fusion proteins were successfully expressed using a prokaryotic system. Notably, both tandem proteins exhibited enhanced immunoreactivity compared to their individual components. This finding is consistent with previous reports: among the structural proteins, VP2 showed superior immunoreactivity over VP1 and VP3 [14], and for the NSPs, 3AB itself was more immunoreactive than 3C [25].

The microarray showed high analytical sensitivity and specificity, as confirmed by performance validation against the VNT. Antibodies against the VP2‐VP3‐VP1 tandem antigen seroconverted at a similar time to neutralizing antibodies, supporting its utility for immune monitoring. In contrast, antibodies against the 3AB‐3C tandem were detected exclusively in sera from naturally infected animals and were absent in those immunized with an inactivated vaccine, confirming its reliable DIVA functionality. This study validates the DIVA concept for inactivated whole‐virus vaccines. Future work should evaluate its applicability to animals immunized with other platforms, such as subunit or viral‐vectored vaccines, as these may elicit distinct antibody responses to NSPs. It is noteworthy that although the 3AB‐3C assay showed higher analytical sensitivity (detection up to a 1:32 dilution) than the VP2‐VP3‐VP1‐based assay (1:16 dilution), its seroconversion was markedly delayed compared to antibodies against structural proteins. Supporting this pattern, our prior work had already established via indirect ELISA that anti‐3AB antibody seroconversion occurs later than that of neutralizing antibodies [33], further substantiating the inherent kinetic differences between humoral responses to structural and non‐structural antigens. The differing kinetics of these responses dictate their separate clinical applications: the VP2‐VP3‐VP1 antigen is optimal for early infection screening and vaccination monitoring, whereas the 3AB‐3C antigen is particularly valuable for retrospective serological surveillance and confirming natural infection in DIVA strategies.

## 5. Conclusion

This study successfully establishes a novel SVA protein microarray as a reliable serological assay for DIVA. The assay exhibits a 97.5% concordance with the VNT and differentiates infected from vaccinated animals by simultaneously detecting antibodies against structural and NSPs. Moreover, its capacity for early detection of seroconversion to structural protein tandem highlights its value as a sensitive diagnostic marker. These findings collectively validate the microarray as a high‐throughput platform for accurately assessing SVA‐specific immune responses in pigs, thereby significantly enhancing vaccine efficacy evaluation and outbreak monitoring.

## Author Contributions

Kegong Tian and Xiangdong Li contributed to the conception of the study, supervision, project administration, funding acquisition, and reviewing. Dexin Li, Junhua Deng, and Zenglin Wang contributed to the methodology, investigation, software use, data curation, visualization, and the preparation of the original draft. Yunjing Zhang, Yufang Li, and Liying Hao contributed to the methodology and investigation.

## Funding

This study was supported by the National Key Research and Development Program of China (Grant 2023YFD1800500), the Postgraduate Research & Practice Innovation Program of Jiangsu Province (Grant SJCX25_2370), the 111 Project D18007, and the Priority Academic Program Development of Jiangsu Higher Education Institutions (PAPD).

## Disclosure

All authors have read and agreed to the published version of the manuscript.

## Conflicts of Interest

Luoyang Putai Biotechnology Co., Ltd. provided experimental materials. Although the company has potential commercial interests in veterinary diagnostics, it had no role in study design, data interpretation, manuscript preparation, or the decision to publish. The authors therefore declare no substantive conflicts of interest regarding the research outcomes.

## Supporting Information

Additional supporting information can be found online in the Supporting Information section.

## Supporting information


**Supporting Information** **Figure S1:** Establishment of the protein microarray based on individual SVA structural (VP1, VP2, VP3) and non‐structural (3AB, 3C) proteins. **Table S1:** Primers used for constructs.

## Data Availability

The data are available upon request from the authors.
