# Supplementary material for: Establishment and Application of a Novel Protein Microarray for Serological Detection and Differentiation of Senecavirus A
Source: Transbound Emerg Dis. 2026 Feb 3;2026:5543555. doi: 10.1155/tbed/5543555 (PMC12867089; doi:10.1155/tbed/5543555)
Supplement: Supplementary file 1 — Supporting Information Figure S1: Establishment of the protein microarray based on individual SVA structural (VP1, VP2, VP3) and non‐structural (3AB, 3C) proteins. Table S1: Primers used for constructs. [file TBED-2026-5543555-s001.docx]

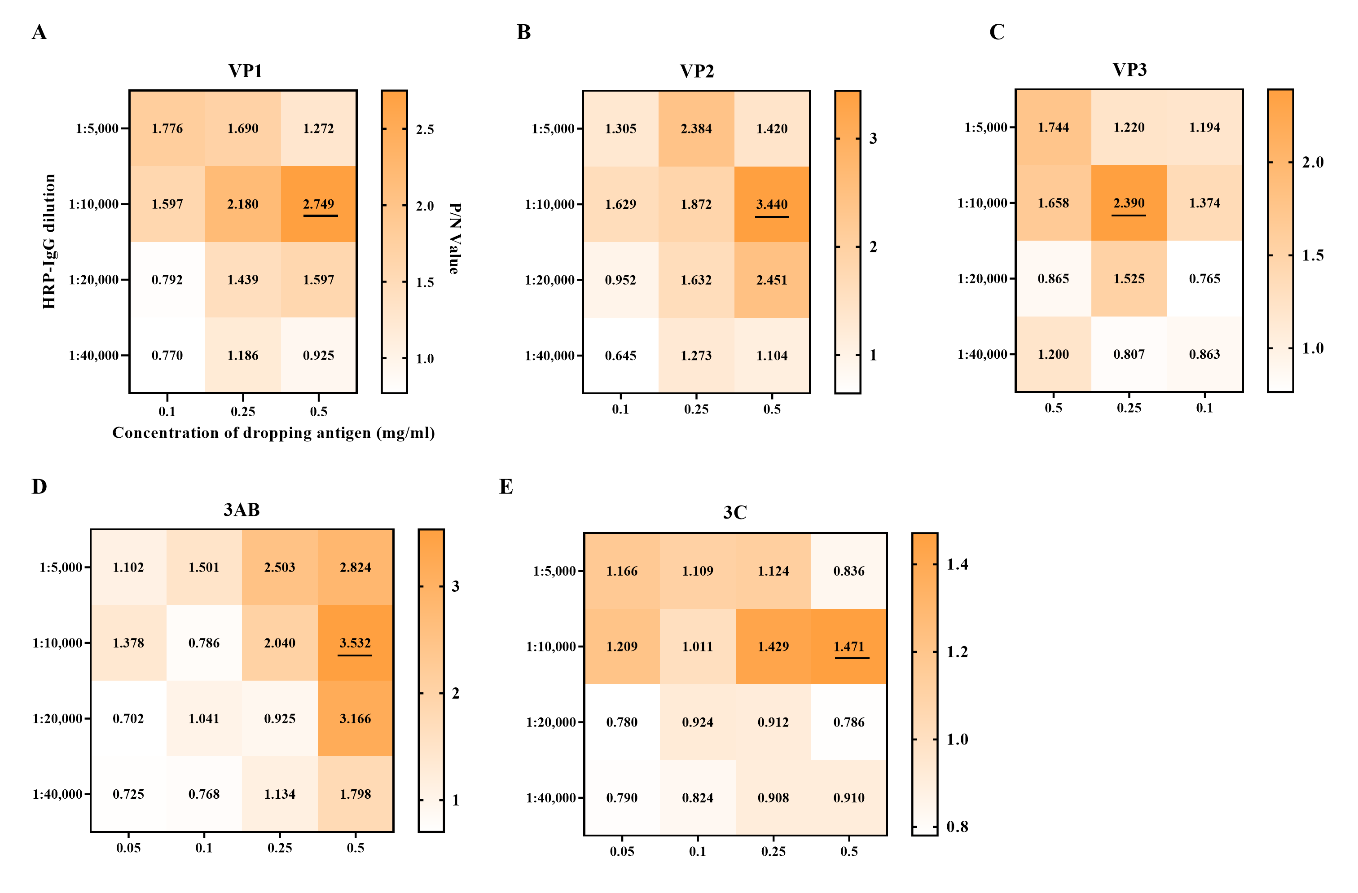


Figure S1 Establishment of the protein microarray based on individual SVA structural (VP1, VP2, VP3) and non-structural (3AB, 3C) proteins. (A−E) Checkerboard titration determined the optimal antigen spotting concentration and secondary antibody dilution for the recombinant VP1, VP2, VP3, 3AB, and 3C proteins. Heatmaps (generated with GraphPad Prism) display positive-to-negative (P/N) ratios. The line graph represents the corresponding P/N ratios.

Table S1: Primers used for constructs.

| Constructs | Primer (5′-3′) | Vector | Insoluble/ Soluble |
| --- | --- | --- | --- |
| pCold-I/ pCold-TF | F: GAGCTCGGTACCCTCGAGGG |  |  |
|  | R: CATATGCCTACCTTCGATATGATGA |  |  |
| pET-28a | F: CGTCGACAAGCTTGCGGC |  |  |
|  | R: GAGCTCGAATTCGGATCCG |  |  |
| VP1 | F: ^1^atatcgaaggtaggcatatgATGTCCACCGACAACGCCGAGACTG | pCold-TF | Soluble |
|  | R: ccctcgagggtaccgagctcTTATTGCATCAGCATCTTCTGCTTG |  |  |
| VP2 | F: gcggatccgaattcgagctcATGGATCGAGTCATAACACAAACG | pET-28a | Insoluble |
|  | R: cggccgcaagcttgtcgacgCCCGGTCTTGAAACGGTTACG |  |  |
| VP3 | F: atatcgaaggtaggcatatgATGGGGCCCATTCCCACAGCACCCA | pCold-I | Insoluble |
|  | R: ccctcgagggtaccgagctcTTAGTGGAACACGTAGGAAGGATTA |  |  |
| 3AB | F: atatcgaaggtaggcatatgATGAGCCCTAATGAGAACGACGACA | pCold-TF | Soluble |
|  | R: ccctcgagggtaccgagctcTTATTGCATTTCCATAAGAGAGAGC |  |  |
| 3C | F: atatcgaaggtaggcatatgATGCAGCCCAACGTGGACATGGGCT | pCold-I | Soluble |
|  | R: ccctcgagggtaccgagctcTTATTGCATTGTAGTCAGAGGCTCA |  |  |
| VP2-VP3-VP1 | ^2^/ | pCold-I | Insoluble |
|  | / |  |  |
| 3AB-3C | / | pCold-I | Soluble |
|  | / |  |  |

Note: ^1^ Lowercase letters represent the homologous arms for vector recombination; ^2^ The symbol “/” indicates that no primer was required.

**Table Caption:** This table presents the primer sequences, vectors, and solubility of the expressed proteins for this study, with solubility categorized as "Soluble" (expression in the supernatant) or "Insoluble" (expression as inclusion bodies).
